# Simple surrogate equations to predict controlled attenuation parameter values for screening non-alcoholic fatty liver disease in a Chinese population

**DOI:** 10.3389/fmed.2022.894895

**Published:** 2022-07-22

**Authors:** Hanying Liu, Xiao Li, Xiaodong Han, Yan Zhang, Yanting Gu, Lianjie Sun, Junfeng Han, Yinfang Tu, Yuqian Bao, Wenkun Bai, Haoyong Yu

**Affiliations:** ^1^Department of Endocrinology and Metabolism, Shanghai Jiao Tong University Affiliated Sixth People’s Hospital, Shanghai Diabetes Institute, Shanghai Clinical Center of Diabetes, Shanghai Key Laboratory of Diabetes Mellitus, Shanghai Key Clinical Center for Metabolic Disease, Shanghai, China; ^2^Department of Ultrasound in Medicine, Shanghai Jiao Tong University Affiliated Sixth People’s Hospital, Shanghai Institute of Ultrasound in Medicine, Shanghai, China; ^3^Department of General Surgery, Shanghai Jiao Tong University Affiliated Sixth People’s Hospital, Shanghai, China

**Keywords:** non-alcoholic fatty liver disease, transient elastography, controlled attenuation parameter, prediction equation, FibroScan

## Abstract

**Objective:**

Non-alcoholic fatty liver disease (NAFLD) is one of the leading causes of chronic liver disease. The controlled attenuation parameter (CAP) obtained by FibroScan reflects the level of liver steatosis in patients with obesity. Our study aimed to construct a simple equation to predict the CAP, to facilitate the screening and monitoring of patients at high risk for NAFLD.

**Methods:**

A total of 272 subjects were randomly divided into derivation and validation cohorts at a ratio of 1:2. The derivation set was used for constructing a multiple linear regression model; the validation set was used to verify the validity of the model.

**Results:**

Several variables strongly correlated with the CAP were used to construct the following equation for predicting CAP values:CAP1 = 2.4 × BMI + 10.5 × TG+ 3.6 × NC + 10.3 × CP +31.0, where BMI is body mass index, TG is triglyceride, NC is neck circumference and CP is C-peptide. The CAP1 model had an *R*^2^ of 0.764 and adjusted *R*^2^ of 0.753. It was then simplified to derive CAP2 included only simple anthropometric parameters: CAP2 = 3.5 × BMI + 4.2 × NC + 20.3 (*R*^2^ = 0.696, adjusted *R*^2^ = 0.689). The data were well fitted by both models. In the verification group, the predicted (CAP1 and CAP2) values were compared to the actual CAP values. For the CAP1 equation, *R*^2^ = 0.653, adjusted *R*^2^ = 0.651. For the CAP2 equation, *R*^2^ = 0.625, adjusted *R*^2^ = 0.623. The intra-class correlation coefficient (ICC) values were 0.781 for CAP1 and 0.716 for CAP2 (*p* < 0.001). The actual CAP and the predicted CAP also showed good agreement in Bland-Altman plot.

**Conclusion:**

The equations for predicting the CAP value comprise easily accessible variables, and showed good stability and predictive power. Thus, they can be used as simple surrogate tools for early screening and follow-up of NAFLD in the Chinese population.

## Introduction

Non-alcoholic fatty liver disease (NAFLD) is one of the major causes of chronic liver disease, with a prevalence of 25–30% in the general population, increasing to 70–90% in the morbidly obese (1–3). NAFLD is linked to a higher risk of cardiovascular disease (CVD), insulin resistance (IR), type 2 diabetes (T2D), obesity, chronic kidney disease (CKD), and other complications (2, 4, 5). Clinical phenotypes of NAFLD range from simple steatosis (non-alcoholic fatty liver) to non-alcoholic steatohepatitis (NASH), advanced fibrosis, cirrhosis and hepatocellular carcinoma (6). Early NAFLD detection, diagnosis, and intervention may reduce the incidence of liver cirrhosis, hepatocellular carcinoma, and other adverse outcomes. For NAFLD diagnosis, liver biopsy remains the “gold standard”. Liver histology is mostly characterized by simple steatosis among NAFLD patients, whereas up to 30% of patients exhibit liver inflammation or fibrosis. It has also been reported that even simple steatosis is not prognostically as “benign” as previously thought and is associated with an increased risk of all-cause mortality (6). This suggests the need for screening of simple steatosis. However, liver biopsy is impractical and unsuitable for widespread clinical screening and follow-up application due to its complications, invasiveness, poor reproducibility and potential for sampling errors.

Currently, routinely used modalities (laboratory tests, ultrasound, computed tomography, and magnetic resonance imaging) are inadequate for determining the levels of liver steatosis. Transient elastography (TE; e.g., FibroScan), a new radiological imaging technique, is reportedly a non-invasive, accurate and reliable method to obtain the controlled attenuation parameter (CAP) generated by proprietary algorithms based on ultrasonic attenuation coefficients to assess the degree of liver steatosis in patients with NAFLD. Despite multiple studies have demonstrated good and acceptable diagnostic accuracy of CAP to quantify steatosis (3, 7–12), it is not a gold standard currently since there is no international consensus on optimal cut-offs. It has also been demonstrated that the method has the advantages of non-invasiveness, good repeatability and reliability, and also covers a liver volume 100 larger than liver biopsy, allowing for population-wide screening of NAFLD (7). Using CAP measurement as a screening method, a substantial number of patients may avoid liver biopsy consequently (7). Therefore, although the method requires further validation, it could be useful in clinical practice as first-line tool to detect patients with NAFLD to help determine those who may still require a liver biopsy (13).

However, as an emerging tool for non-invasive assessment of NAFLD, FibroScan is not widely applied in all hospitals and primary medical care centers. Thus, our study aimed to identify the clinical variables associated with hepatic steatosis that are strongly correlated with the CAP value, to derive a simple equation for predicting the CAP value to facilitate screening and follow-up evaluation in medical institutions with limited FibroScan capability.

## Subjects and methods

### Subjects

Our study conducted academic enrollment from Jul 2020 to Feb 2021 at Shanghai Jiao Tong University Affiliated Sixth People’s Hospital, China. Subjects were included from obesity outpatient clinic and regular physical examination center in the hospital, aged 16-60 years. Exclusion criteria of our study included: (i) excessive alcohol consumption (>140 g/week for men and >70 g/week for women),(ii) suffering from hepatitis B or hepatitis C infection, (iii) current malignancy with a life expectancy less than 6 months, (iv) taking medications known to precipitate fatty liver such as estrogens, tamoxifen, amiodarone, methotrexate, and glitazones (3) during the previous 6 months, (v) suffering from autoimmune liver disease or inherited metabolic storage disorders. A total of 272 subjects who underwent valid FibroScan examinations were recruited, including 77 males and 195 females. There were 192 obese subjects and 80 non-obese subjects among them in terms of body mass index (BMI).CAP, anthropometric measurements, and laboratory assessments were performed in all participants. Then, they were randomly assigned to derivation and validation cohorts at a ratio of 1:2 for the construction and verification of the model. The study protocol was approved by the Ethics Review Committee of Shanghai Jiao Tong University Affiliated Sixth People’s Hospital. All procedures followed were in accordance with the ethical standards of the responsible committee on human experimentation and with the Helsinki Declaration of 1975, as revised in 2008. All respondents provided informed consent and participated voluntarily.

### Controlled attenuation parameter measurement

All the CAP measurements were completed by an experienced operator on a FibroScan touch 502 (Echosens, Paris, France) using XL probe (3). During the examination, subjects lied supine and elevated the right hand with their right rib cage spread (3, 7). The probe was placed vertically on the skin surface between the 9th to 11th intercostal spaces on the mid-axillary line or the anterior axillary line adjacent to the right lobe of the liver (7). The range of the liver detected was located in the subcutaneous 2.5∼6.5 cm and there was no macrovascular structure. The inter quartile range (IQR) was an indicator of the variance of CAP values defined as the interval containing 50% of the valid measurements between the 25th to 75th percentiles. The ratios of the IQR and median values (IQR/M) of the CAP were calculated to test the variability. At least ten successful acquisitions were performed on each subject. The measurement was considered reliable if the IQR/M of CAP obtained from ten valid acquisitions was less than 0.3 (3, 14). The operator of FibroScan was blinded to the clinical and biological information of the subjects. Based on the standard of CAP cut-offs proposed by Sasso et al. (10), which is the uniform standard used in China, CAP value of ≥238 dB/m indicates the presence of fatty liver (7, 10).

### Clinical and laboratory assessments

The height and weight of participants wearing light clothes were measured by a digital scale to calculate BMI = body weight (kg)/ height squared (m^2^). Waist circumference (WC) was measured by a trained examiner on the midline between the lower border of the rib cage and the anterior superior iliac spine. Then, the subjects stood upright with legs close together, and the tape was placed horizontally over their anterior pubic symphysis and the most convex part of the posterior gluteus maximus to measure the hip circumference (HC). Neck circumference (NC) was measured with head erect and eyes facing forward, horizontally at the upper margin of the laryngeal prominence. Systolic blood pressure (SBP) and diastolic blood pressure (DBP) were measured twice during one day in a quiet state and analyses were performed on mean values.

Laboratory variables included: alanine aminotransferase (ALT), aspartate aminotransferase (AST), γ-glutamyl transpeptidase (γ-GT),alkaline phosphatase (ALP), prealbumin (PAB), total bile acid (TBA), total bilirubin (TBiL), direct bilirubin (DBiL), blood urea nitrogen (BUN), serum creatinine (Scr), serum uric acid (SUA), retinol-binding protein (RBP), and cystatin C (Cys-C),total cholesterol(TC), triglyceride (TG), high-density lipoprotein cholesterol (HDL-c), low-density lipoprotein cholesterol (LDL-c),serum fasting blood glucose(FBG), hemoglobin A1c (HbA1c),insulin, C-peptide (CP). All subjects had a low-fat diet one day before and venous blood samples were taken in the early morning after fasting for 8 hours. All biochemical determinations were performed in the same lab using standard laboratory methods. Insulin was measured with intra- and interassay coefficients of variation <5% by radioimmunoassay (ADVIA Insulin Ready Pack 100, Bayer Diagnostics, Milan, Italy).

### Statistical analysis

All analyses were conducted using SPSS version 26.0 (SPSS Inc., Chicago, IL, USA), and P-values of < 0.05 were considered statistically significant. Continuous variables were compared using the Student’s t-test or the Mann-Whitney U-test and were expressed as mean ± standard deviation (SD) or median (IQR). Categorical variables were compared using the chi-square test or Fisher’s exact test and were expressed as percentages. The relationship between different variables with CAP was analyzed using the Pearson or Spearman correlation. Variables strongly correlated with the CAP (*p* < 0.01, *r* > 0.3) by correlation analysis were introduced into the multiple linear regression model, and then the independent predictors of CAP values were identified and screened out stepwise to construct the prediction equation. Further, the accuracy of the equation was verified on validation set by reliability analysis and Bland–Altman plot.

## Results

### Baseline characteristics

A total of 272 subjects with valid CAP measurements were recruited. There were 192 obese subjects and 80 non-obese subjects, and CAP values of the obese group and the non-obese group were statistically different (*p* < 0.01). CAP values were much higher in the obese group. Then the 272 subjects were divided into derivation and verification groups randomly at a ratio of 1:2, including 91 in the derivation cohort and 181 patients enrolled in a separate cohort as the validation set. Table 1 gives the demographic and clinical characteristics of the subjects in two cohorts. No statistically significant difference was observed between them in terms of baseline characteristics (p > 0.05).

**TABLE 1:** Baseline characteristics in the derivation and validation cohorts.

Characteristics	Derivation cohort	Validation cohort	*P*-value
N (M/F)	91(26/65)	181(51/130)	0.95
Age (years)	31.4 ± 8.2	31.1 ± 7.0	0.73
CAP (dB/m)	320.0(255.0, 366.0)	335.0(267.5, 376.0)	0.11
BMI (kg/m^2^)	34.1 ± 10.2	33.9 ± 8.7	0.85
WHR	1.0(0.9, 1.0)	1.0(0.9, 1.0)	0.88
HR	82.0(77.8, 91.3)	85.0(77.0, 95.5)	0.32
SBP (mmHg)	126.0(115.8, 136.0)	125.0(115.0, 136.5)	0.87
DBP (mmHg)	82.0(73.8, 91.3)	84.0(76.0, 91.0)	0.28
NC (cm)	39.0(35.5, 42.5)	38.5(36.0, 41.3)	0.84
ALT (U/L)	31.0(18.0, 65.0)	33.0(22.0, 69.5)	0.29
AST (U/L)	22.0(17.0, 36.0)	22.0(18.0, 38.0)	0.62
γ-GT(U/L)	28.0(16.0, 48.0)	30.0(18.0, 49.5)	0.79
ALP (U/L)	67.0(54.0, 83.0)	69.0(58.0, 84.5)	0.47
PAB (mg/L)	277.0(235.0, 313.0)	274.5(244.0, 301.8)	0.85
TBA (μmol/L)	4.0(2.3, 7.3)	3.4(2.3, 5.4)	0.11
TBiL (μmol/L)	8.8(6.2, 11.5)	9.1(6.7, 12.3)	0.36
DBiL (μmol/L)	2.6(1.9, 3.3)	2.4(1.8, 3.4)	0.34
BUN (mmol/L)	4.6(3.9, 5.6)	4.5(3.7, 5.1)	0.23
Scr (mg/dL)	62.0(53.0, 72.0)	60.0(53.0, 71.5)	0.67
SUA (mg/dL)	398.9 ± 106.6	383.1 ± 113.9	0.27
RBP (mg/L)	33.0(29.0, 44.0)	35.0(30.0, 41.0)	0.39
Cys-C(mg/L)	0.7(0.6, 0.8)	0.7(0.6, 0.8)	0.34
TC(mmol/l)	5.1 ± 1.0	5.1 ± 1.0	0.62
TG(mmol/l)	1.2(0.8, 1.7)	1.4(1.0, 2.1)	0.06
HDL-c (mmol/l)	1.3(1.2, 1.5)	1.3(1.2, 1.5)	0.89
LDL -c (mmol/l)	3.0(2.4, 3.6)	3.0(2.5, 3.7)	0.31
FBG(mmol/l)	5.1(4.7, 6.0)	5.4(4.7, 6.4)	0.36
HbA1c (%)	5.6(5.3, 6.5)	5.7(5.4, 6.5)	0.28
Insulin(μIU/ml)	21.1(10.4, 34.6)	20.8(11.2, 35.4)	0.84
CP (ng/ml)	3.5(2.1, 4.6)	3.4(2.2, 4.5)	0.96

Data are expressed as the mean ± SD or the median (inter quartile range); categorical variables are expressed as n, (%).

M/F = male/female, CAP = controlled attenuation parameter, BMI = body mass index, WHR = waist-to-hip ratio, HR = heart rate, SBP = systolic blood pressure, DBP = diastolic blood pressure, NC = neck circumference, ALT = alanine aminotransferase, AST = aspartate aminotransferase, γ-GT = γ-glutamyl transpeptidase, ALP = alkaline phosphatase, PAB = prealbumin, TBA = total bile acid, TBiL = total bilirubin, DBiL = direct bilirubin, BUN = blood urea nitrogen, Scr = serum creatinine, SUA = serum uric acid, RBP = retinol-binding protein, Cys-C = cystatin C, TC = total cholesterol, TG = triglyceride, HDL-c = high-density lipoprotein cholesterol, LDL-c = low-density lipoprotein cholesterol, FBG = fasting blood glucose, HbA1c = glycated hemoglobin, CP = C-peptide.

### Correlation analysis

Bivariate correlation analysis was performed to identify the variables associated with the CAP values among the multiple variables mentioned above. 19 clinical variables correlated with CAP (*r* > 0.3, *p* < 0.01) (Table 2) were obtained. Then we plotted scatter plots of each independent variable against the dependent variable (CAP) separately, found a linear relationship between the CAP and each of the independent variables. Thus these variables were further introduced into the stepwise multiple linear regression model.

**TABLE 2:** The correlation analysis of CAP and 19 clinical variables.

Variable	r	*P*-value
BMI	0.80	0.00
WHR	0.76	0.00
HR	0.41	0.00
SBP	0.53	0.00
DBP	0.47	0.00
NC	0.79	0.00
ALT	0.56	0.00
AST	0.51	0.00
γ-GT	0.57	0.00
ALP	0.46	0.00
SUA	0.46	0.00
Cys-C	0.56	0.00
TG	0.57	0.00
HDL-c	−0.39	0.00
LDL-c	0.31	0.00
FBG	0.42	0.00
HbA1c	0.42	0.00
Insulin	0.70	0.00
CP	0.73	0.00

BMI = body mass index, WHR = waist-to-hip ratio, HR = heart rate, SBP = systolic blood pressure, DBP = diastolic blood pressure, NC = neck circumference, ALT = alanine aminotransferase, AST = aspartate aminotransferase, γ-GT = γ-glutamyl transpeptidase, ALP = alkaline phosphatase, SUA = serum uric acid, Cys-C = cystatin C, TG = triglyceride, HDL-c = high-density lipoprotein cholesterol, LDL-c = low-density lipoprotein cholesterol, FBG = fasting blood glucose, HbA1c = glycated hemoglobin, CP = C-peptide.

### Equation development

In the multiple linear regression model, four variables strongly correlated with the CAP were screened out to construct an equation for predicting the CAP value (stepwise, *p* < 0.01):

CAP1=⁢2.4×BMI+10.5×TG+3.6×NC+10.3×CP+31.0

The model based on the four predictors fitted well with an R^2^ of 0.764 and an adjusted R^2^ of 0.753. Table 3 provides the regression coefficient and 95% confidence interval (CI) of each variable. The Durbin-Watson test of model residuals was 1.803, indicating that the observed CAP values were mutually independent, i.e., there was no significant correlation between the residuals. Based on the collinearity analysis, there was no covariance among the four independent variables. The tolerances were more than 0.2 and the variance inflation factor (VIF) values were less than 5.

**TABLE 3:** The performance of these equations in derivation cohort.

Equation	R^2^	Adjusted R^2^	Durbin-Watson test	Variables	Coefficients	95%CI	*P*-value	Tolerance	VIF
CAP1	0.764	0.753	1.803	BMI	2.40	0.92, 3.88	0.002	0.223	4.478
				TG	10.53	1.30, 19.76	0.026	0.747	1.339
				NC	3.61	1.13, 6.09	0.005	0.273	3.663
				CP	10.26	3.29, 17.24	0.004	0.466	2.147
				Constant	30.97	−31.68, 93.62	0.328	/	/
CAP2	0.696	0.689	1.628	BMI	3.53	2.04, 5.02	0.000	0.279	3.583
				NC	4.20	1.47, 6. 92	0.003	0.279	3.583
				Constant	20.30	−48.81, 89.41	0.561	/	/

BMI = body mass index, TG = triglyceride, NC = neck circumference, CP = C-peptide.

**FIGURE 1:**
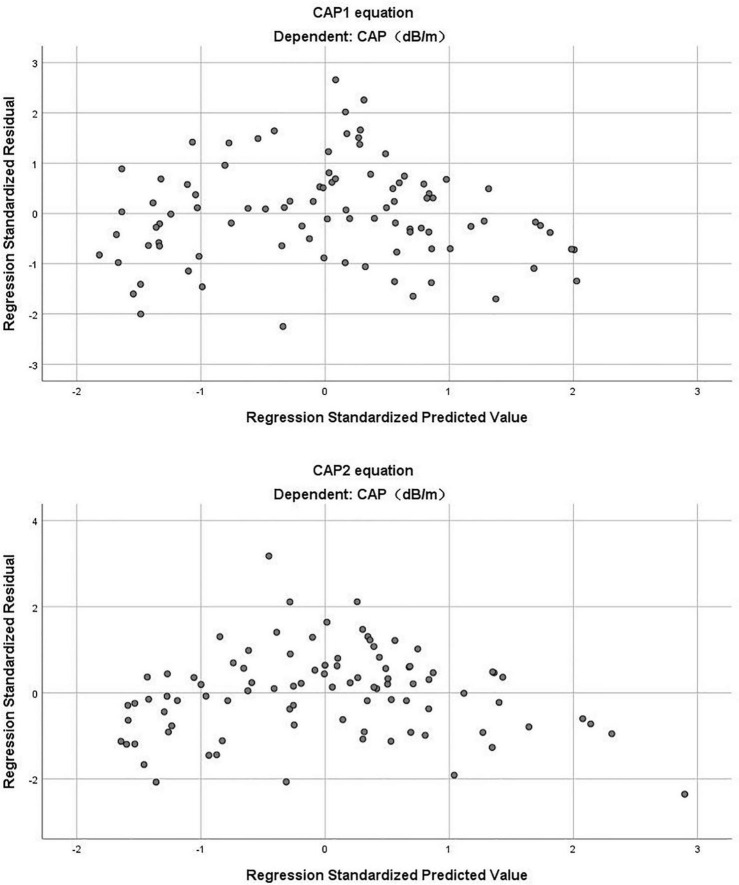
The residual scatter plot of standardized predicted value and standardized residual in CAP1 and CAP2 equation.

**FIGURE 2:**
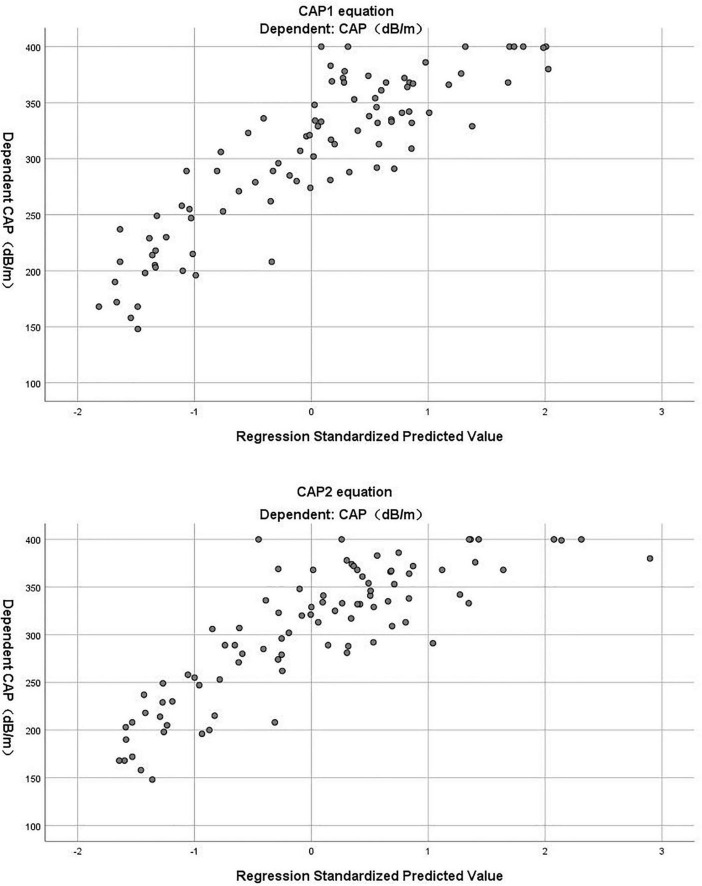
The scatter plot of standardized predicted value and dependent CAP in CAP1 and CAP2 equation.

However, the equation is still relatively complicated and inconvenient to be applied in daily medical examinations, especially for those primary medical care centers where CP and TG serological tests are unconditional to be performed. Therefore, we simplified models further by removing those two variables, only using simple and accessible anthropometric parameters to derive a new set of the equation eventually:

CAP2=3.5×BMI+4.2×NC+20.3

(*R*^2^ = 0.696, adjusted *R*^2^ = 0.689) (Table 3). The Durbin-Watson test of model residuals was 1.628, the tolerance between the independent variables was more than 0.2 and the VIF values were less than 4. Likewise, the model fitted well. In Figure 1, we depicted a residual scatter plot with the standardized predicted value on the *X* axis and the standardized residual on the *Y*-axis. The scatter points had a random distribution, and the slope was close to zero, indicating that there was no auto-correlation observed in both models. Figure 1 showed the variance homogeneity of the residuals. In addition, on the basis of the scatter plot of the standardized predicted value and dependent CAP (Figure 2), there was a linear trend in both equations. We also observed that the residuals were approximately normally distributed through the histograms and normal P-P plots of the residuals. Additionally, the results of the Casewise Diagnostics test (3 SD) were not output by SPSS, indicating there were no significant outliers in our study.

### Verification of equations

**FIGURE 3:**
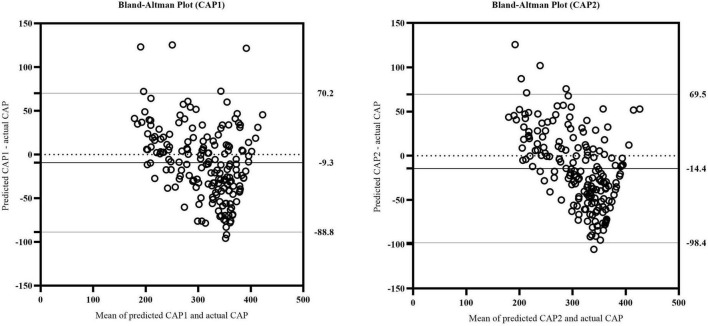
The Bland-Altman plot of actual CAP and predicted CAP in CAP1 and CAP2 equation. The upper and lower horizontal solid lines represented the 95% limits of agreement. The middle horizontal solid line represented the mean value of the differences between predicted and actual CAP. The horizontal dotted line indicated the position where the mean value of the differences was zero.

We further evaluated whether the equations could predict the CAP values as measured by TE. The validation cohort was used to verify the accuracy of the equations. The predicted (CAP1 and CAP2) values were compared to the actual CAP values, respectively, to verify the effectiveness of the models. For the CAP1 equation, *R*^2^ was 0.653 and the adjusted *R*^2^ was 0.651. For the CAP2 equation, *R*^2^ was 0.625 and the adjusted *R*^2^ was 0.623. Reliability analysis was performed to determine the consistency between predicted and actual CAP on the same subject. For the absolute agreement type in two-way random model, the values of intra-class correlation efficient (ICC) (single measures) were 0.781 for CAP1 (95% CI: 0.712-0.834, *p* < 0.001) and 0.716 for CAP2 (95% CI: 0.611-0.792, *p* < 0.001). After plotting a Bland–Altman plot (Figure 3), we observed that the differences between predicted and actual CAP were mostly within the 95% limits of agreement in both equations. Additionally, the mean value of the differences was nearly zero. Therefore, it can be assumed that the predicted CAP exhibited a significant and high consistency with the actual CAP in both equations.

## Discussion

TE is a novel, non-invasive diagnostic technique (unlike invasive liver biopsy) developed recently that can obtain the CAP quickly and quantify the degree of hepatic steatosis. Thus, fatty liver can be screened in a timely manner, allowing for early intervention. The ability of the CAP to detect patients with fatty liver helps identify low-risk patients with NAFLD for whom liver biopsy is unnecessary, thereby reducing the risk of complications, as well as the financial costs (7). However, large-scale screening remains cost-prohibitive and difficult to carry out; thus, an easy and non-invasive method is required to screen patients at high risk of NAFLD. To this end, assessment equations that provide simple, easily measurable, and accessible markers of hepatic steatosis could be used as an alternative of the expensive and complex FibroScan examination to provide a rough estimate of the CAP value. The final equations may serve as preliminary screening tools that allow patients with low risk of NAFLD to avoid liver biopsy, while patients at high risk may still require further biopsy to clarify the pathological diagnosis of NAFLD.

A range of factors reportedly influence the CAP in NAFLD. A prospective research indicated that the CAP value increases significantly in the presence of metabolic syndrome (MetS), arterial hypertension and T2DM, as well as with BMI and WC (15). Garg et al. suggested that age, BMI, the Homeostatic Model Assessment for Insulin Resistance index (HOMA-IR), and the LDL/HDL ratio are independent predictors of the CAP (3). We collected anthropometric and laboratory data from 272 subjects and included strongly correlated variables (p < 0.01, *r* > 0.3) in our stepwise multiple linear regression analysis, revealed that BMI, TG, NC, and CP were independent predictors of the CAP.

BMI is one of the most commonly used indicators of obesity. Several studies have shown that obesity is a significant risk factor for NAFLD. The prevalence of NAFLD in obese individuals is reported as high as 80% compared to only 16% in those with a normal BMI and no metabolic risk factors (2). It has been reported that obese individuals have a 3.5-fold higher risk of developing NAFLD, and BMI is obviously associated with NAFLD risk (RR = 1.20 per unit increment in BMI; 95% CI, 1.14–1.26, *P* < 0.001) (16). Likewise, Pang et al. discovered that the incidence risk of NAFLD increases 0.25-fold with each unit increase in BMI, and is 1.85-fold greater in individuals with a high BMI (17). In previous meta-analyses, metabolism-related serological indicators were significantly higher in obese than lean NAFLD patients (BMI < 25 kg/m^2^), and obese NAFLD patients also had a higher fibrosis score, NAFLD activity score (NAS), and risk of NASH (18). Additionally, obesity can increase the severity of NAFLD, suggesting that it may lead to a worse long-term prognosis in NAFLD (19). Adipocyte death and cytokine secretion are triggered by increased burden of lipid accumulation, resulting in chronic inflammation in obese patients. Chronic inflammation plays a key role on progression of NAFLD. Consequently, obese NAFLD patients tend to have a higher fibrosis score and NAS, as well as an increased risk of hepatic steatosis and metabolic complications, as compared to those lean NAFLD patients (19).

Dyslipidemia is a consistent hallmark of NAFLD. All types of lipoprotein particles are produced and cleared by the liver, thus making it crucial for lipoprotein metabolism. The metabolic dysfunction of the liver in NAFLD is closely related to alteration in lipoprotein metabolism and composition (20). Hepatocytes normally contain about 4–7% total lipids, of which about half is TG (21). The accumulation of TG in liver cells is an essential step in the development of NAFLD. Physiologically, TG concentrations in the liver remain low steady-state resulting from the delicate equilibrium between the accumulation and disposal of TG. Under conditions of overnutrition and IR, multiple factors contributing to the elevated TG concentrations disrupt the balance and ultimately lead to liver steatosis (22). Li et al. found that the NAFLD group has a higher average TG level than the non-NAFLD; thus, TG is related to NAFLD in normal-weight individuals (21). A cross-sectional study conducted by Fan et al. confirmed the independent association between TG/HDL-C and the risk of NAFLD in apparently healthy individuals (23). Patients with normal TG levels may fail to recognize the occurrence and severity of NAFLD, leading to the development of underlying liver lesions and disease progression without proper monitoring. Therefore, monitoring of NAFLD in normal TG patients should be made a high priority to facilitate early intervention.

Neck circumference is a novel anthropometric measure reflecting ectopic fat distribution in the neck, and has proven to be an easily accessible and replicable method for screening patients at risk for NAFLD development (24–26). NC is also associated with known cardiometabolic risk factors, such as IR and abdominal adiposity (25, 27). Ectopic fat distribution, including visceral adiposity, is important risk factor for premature atherosclerosis, the main co-morbidities of NAFLD (28). Interleukin (IL)-17 is the main pro-inflammatory cytokine regulating local tissue inflammation. The IL-17 produced by the macrophages of visceral adipose tissue induces the smooth muscle cells present in the atherosclerotic vessels to secrete eotaxin. Thus, the amount of visceral adiposity and the level of circulating eotaxin are highly predictive of early atherosclerosis (29). It is well known that WC measurement is an extremely simple and inexpensive method used in the clinical setting that strictly relates to visceral adiposity (30, 31). Jia et al. performed a receiver operating characteristic curves analysis indicating that WC has the best accuracy in predicting visceral obesity in comparison with BMI and waist-to-hip ratio (WHR) (32). However, WC evaluation is limited by several factors. First, there are various ways to measure WC from different anatomical landmarks in clinical studies, influencing the absolute WC values obtained. Second, WC varies throughout the day, and is influenced by the abdominal organs/cavity and the structure of the abdominal wall, e.g., severe obesity, abdominoplasty, or major weight loss. Third, since the evaluation requires undressing, WC measurement is more difficult to perform particularly in cold weather (24). NC measurement is more feasible than WC for large-scale population studies. It has an explicit anthropometry landmark requiring only one anthropometrist and a tape (25). The low variability minimally affected by breathing and diet making NC easier and highly reproducible to measure (26). Luo et al. provided the NC cut-offs for identifying visceral adiposity : ≥38.5 cm with a sensitivity of 56.1% and specificity of 83.5% for men and ≥34.5 cm with a sensitivity of 58.1% and specificity of 82.5% for women. Moreover, they demonstrated that there are no differences in the sensitivity and specificity between NC and WC for the diagnosis of metabolic disorders (33). Peña-Vélez et al. observed that every 1 cm increase in NC leads to a 19% higher risk of NAFLD (25). Another study revealed that NAFLD risk increases 1.74-fold for men and 1.73-fold for women with every 1 cm of NC (34). The mechanism underlying the association between NC and NAFLD is being actively researched.

Previous studies showed that there is an association between CP and NAFLD in specific groups (e.g., diabetics and obese individuals) as well as in the general population. Atsawarungruangkit et al. demonstrated that CP is superior to fasting insulin levels for screening or monitoring IR in NAFLD (35). Similarly, Han et al. suggested that obese children with high levels of fasting CP are at increased risk for developing NAFLD; thus, CP has significant predictive value with respect to NAFLD onset (36). Notably, Wang et al. observed that CP levels are significantly associated with NAFLD inflammatory and fibrotic progression (37). It is helpful to understand islet hormone changes associated with T2D, and to differentiate among NAFLD stages for more effective, personalized treatment of NAFLD patients with T2D. The role of CP in the progression of inflammation and steatosis in NAFLD warrants further investigation.

In our study, we further developed a simplified equation to determine the CAP value using only readily accessible anthropometric parameters for use in primary medical centers and community hospitals with limited laboratory access. This auxiliary method is simple and reliable, thus allowing for population-wide screening and monitoring of NAFLD.

According to what we discussed above, we could prevent the occurrence or delay the development of NAFLD by early detection and active interventions. For NAFLD, the ideal drug candidate should ameliorate steatosis, inflammation and fibrosis, as well as glucose metabolism, IR and obesity (38). A variety of pharmacological approaches using existing drugs have been used clinically in the management of NAFLD, including antioxidants, antidiabetic, anti-obesity, and cytoprotective agents (39). Currently, there are various potential pharmacological targets and emerging therapeutics with specific interventional mechanisms: (i) metabolic targeted therapies, (ii) oxidative stress targeted therapies, (iii) inflammation targeted therapies, (iv) apoptosis targeted therapies, and (v) fibrosis targeted therapies (39). In addition to monotherapy, a number of combination therapies targeting multiple targets or mechanisms are being investigated in clinical trials (38, 39).

This study had several limitations. First, it was a single-center study; thus, it was difficult to avoid selection bias. The findings in our Asian population cannot be generalized to other ethnic groups. Therefore, the equations require further validation in other ethnic groups and centers. Second, the proportion of males was relatively small; the sample size should be expanded in future studies, with well-balanced study parameters. Third, liver biopsy was not performed in all patients, leading to the inability to compare the model with the gold standard. Further validation of the equation for the diagnosis of NAFLD compared to the gold standard is needed in future studies. Finally, the study was cross-sectional, so more prospective studies are needed to confirm the utility of the equations for identifying and predicting NAFLD development.

## Conclusion

The equations proposed for predicting the CAP value may serve as surrogate tools for early screening and follow-up of NAFLD.

## Data availability statement

The original contributions presented in the study are included in the article/supplementary material, further inquiries can be directed to the corresponding author/s.

## Ethics statement

The studies involving human participants were reviewed and approved by Ethics Committee of Shanghai Jiao Tong University Affiliated Sixth People’s Hospital. The patients/participants provided their written informed consent to participate in this study.

## Author contributions

HL and XL drafted the manuscript. HL and XH performed the statistical analysis. YT and JH drafted the figure and legend. YZ and YG collected ultrasound data. YB, WB and HY designed the outline of the topic and helped on revising the manuscript. All authors read and approved the submitted version, contributed to the study conception and design.

## References

[1] Li J Zou B Yeo YH Feng Y Xie X Lee DH Prevalence, incidence, and outcome of non-alcoholic fatty liver disease in Asia, 1999-2019: a systematic review and meta-analysis. *Lancet Gastroenterol Hepatol.* (2019) 4:389–98. 10.1016/s2468-1253(19)30039-1 30902670

[2] Miliæ S Luliæ D Štimac D. Non-alcoholic fatty liver disease and obesity: biochemical, metabolic and clinical presentations. *World J Gastroenterol.* (2014) 20:9330–7. 10.3748/wjg.v20.i28.9330 25071327 PMC4110564

[3] Garg H Aggarwal S Shalimar, Yadav R Datta Gupta S Agarwal L Utility of transient elastography (fibroscan) and impact of bariatric surgery on nonalcoholic fatty liver disease (NAFLD) in morbidly obese patients. *Surg Obes Relat Dis.* (2018) 14:81–91. 10.1016/j.soard.2017.09.005 29126863

[4] Anstee QM Targher G Day CP. Progression of NAFLD to diabetes mellitus, cardiovascular disease or cirrhosis. *Nat Rev Gastroenterol Hepatol.* (2013) 10:330–44. 10.1038/nrgastro.2013.41 23507799

[5] Lonardo A Sookoian S Chonchol M Loria P Targher G. Cardiovascular and systemic risk in nonalcoholic fatty liver disease - atherosclerosis as a major player in the natural course of NAFLD. *Curr Pharm Des.* (2013) 19:5177–92. 23432668

[6] Tilg H Targher G. Nafld-related mortality: simple hepatic steatosis is not as ‘Benign’ as thought. *Gut.* (2021) 70:1212–3. 10.1136/gutjnl-2020-323188 33077572

[7] Mikolasevic I Orlic L Franjic N Hauser G Stimac D Milic S. Transient elastography (Fibroscan(§)) with controlled attenuation parameter in the assessment of liver steatosis and fibrosis in patients with nonalcoholic fatty liver disease - where do we stand? *World J Gastroenterol.* (2016) 22:7236–51. 10.3748/wjg.v22.i32.7236 27621571 PMC4997649

[8] Ferraioli G Soares Monteiro LB. Ultrasound-based techniques for the diagnosis of liver steatosis. *World J Gastroenterol.* (2019) 25:6053–62. 10.3748/wjg.v25.i40.6053 31686762 PMC6824276

[9] Castera L Friedrich-Rust M Loomba R. Noninvasive assessment of liver disease in patients with nonalcoholic fatty liver disease. *Gastroenterology.* (2019) 156:1264–81.e4. 10.1053/j.gastro.2018.12.036 30660725 PMC7505052

[10] Sasso M Beaugrand M de Ledinghen V Douvin C Marcellin P Poupon R Controlled attenuation parameter (CAP): a novel VCTE™ guided ultrasonic attenuation measurement for the evaluation of hepatic steatosis: preliminary study and validation in a cohort of patients with chronic liver disease from various causes. *Ultrasound Med Biol.* (2010) 36:1825–35. 10.1016/j.ultrasmedbio.2010.07.005 20870345

[11] Sirli R Sporea I. Controlled attenuation parameter for quantification of steatosis: which cut-offs to use? *Can J Gastroenterol Hepatol.* (2021) 2021:6662760. 10.1155/2021/6662760 33834008 PMC8018863

[12] Han MA Saouaf R Ayoub W Todo T Mena E Noureddin M. Magnetic resonance imaging and transient elastography in the management of nonalcoholic fatty liver disease (NAFLD). *Expert Rev Clin Pharmacol.* (2017) 10:379–90. 10.1080/17512433.2017.1299573 28277807 PMC6658175

[13] Castera L Vilgrain V Angulo P. Noninvasive evaluation of NAFLD. *Nat Rev Gastroenterol Hepatol.* (2013) 10:666–75. 10.1038/nrgastro.2013.175 24061203

[14] Oeda S Tanaka K Oshima A Matsumoto Y Sueoka E Takahashi H. Diagnostic accuracy of fibroscan and factors affecting measurements. *Diagnostics (Basel).* (2020) 10:940. 10.3390/diagnostics10110940 33198092 PMC7696616

[15] de Lédinghen V Vergniol J Capdepont M Chermak F Hiriart JB Cassinotto C Controlled attenuation parameter (CAP) for the diagnosis of steatosis: a prospective study of 5323 examinations. *J Hepatol.* (2014) 60:1026–31. 10.1016/j.jhep.2013.12.018 24378529

[16] Li L Liu DW Yan HY Wang ZY Zhao SH Wang B. Obesity is an independent risk factor for non-alcoholic fatty liver disease: evidence from a meta-analysis of 21 cohort studies. *Obes Rev.* (2016) 17:510–9. Epub 2016/03/30. 10.1111/obr.12407., 27020692

[17] Pang Q Zhang JY Song SD Qu K Xu XS Liu SS Central obesity and nonalcoholic fatty liver disease risk after adjusting for body mass index. *World J Gastroenterol.* (2015) 21:1650–62. 10.3748/wjg.v21.i5.1650 25663786 PMC4316109

[18] Sookoian S Pirola CJ. Systematic review with meta-analysis: the significance of histological disease severity in lean patients with nonalcoholic fatty liver disease. *Aliment Pharmacol Ther.* (2018) 47:16–25. 10.1111/apt.14401 29083036

[19] Lu FB Hu ED Xu LM Chen L Wu JL Li H The relationship between obesity and the severity of non-alcoholic fatty liver disease: systematic review and meta-analysis. *Expert Rev Gastroenterol Hepatol.* (2018) 12:491–502. 10.1080/17474124.2018.1460202 29609501

[20] Deprince A Haas JT Staels B. Dysregulated lipid metabolism links NAFLD to cardiovascular disease. *Mol Metab.* (2020) 42:101092. 10.1016/j.molmet.2020.101092 33010471 PMC7600388

[21] Li Y Chen Y Tian X Zhang S Jiao J. Comparison of clinical characteristics between obese and non-obese patients with nonalcoholic fatty liver disease (NAFLD). *Diabetes Metab Syndr Obes.* (2021) 14:2029–39. 10.2147/dmso.S304634 33986604 PMC8110261

[22] Kawano Y Cohen DE. Mechanisms of hepatic triglyceride accumulation in non-alcoholic fatty liver disease. *J Gastroenterol.* (2013) 48:434–41. 10.1007/s00535-013-0758-5 23397118 PMC3633701

[23] Fan N Peng L Xia Z Zhang L Song Z Wang Y Triglycerides to high-density lipoprotein cholesterol ratio as a surrogate for nonalcoholic fatty liver disease: a cross-sectional study. *Lipids Health Dis.* (2019) 18:39. 10.1186/s12944-019-0986-7 30711017 PMC6359827

[24] Li Q Wang N Han B Chen Y Zhu C Chen Y Neck circumference as an independent indicator to non-alcoholic fatty liver disease in non-obese men. *Nutr Metab (Lond).* (2015) 12:63. 10.1186/s12986-015-0060-z 26719755 PMC4696111

[25] Peña-Vélez R Garibay-Nieto N Cal YM-VM Laresgoiti-Servitje E Pedraza-Escudero K García-Blanco MDC Association between neck circumference and non-alcoholic fatty liver disease in mexican children and adolescents with obesity. *J Pediatr Endocrinol Metab.* (2020) 33:205–13. 10.1515/jpem-2019-0204 31846425

[26] Jian C Xu Y Ma X Shen Y Wang Y Bao Y. Neck circumference is an effective supplement for nonalcoholic fatty liver disease screening in a community-based population. *Int J Endocrinol.* (2020) 2020:7982107. 10.1155/2020/7982107 32508918 PMC7246413

[27] Li HX Zhang F Zhao D Xin Z Guo SQ Wang SM Neck circumference as a measure of neck fat and abdominal visceral fat in Chinese adults. *BMC Public Health.* (2014) 14:311. 10.1186/1471-2458-14-311 24708638 PMC4004507

[28] Neeland IJ Ross R Després JP Matsuzawa Y Yamashita S Shai I Visceral and ectopic fat, atherosclerosis, and cardiometabolic disease: a position statement. *Lancet Diabetes Endocrinol.* (2019) 7:715–25. 10.1016/s2213-8587(19)30084-1 31301983

[29] Tarantino G Costantini S Finelli C Capone F Guerriero E La Sala N Is serum Interleukin-17 associated with early atherosclerosis in obese patients? *J Transl Med.* (2014) 12:214. 10.1186/s12967-014-0214-1 25092442 PMC4256548

[30] Ross R Neeland IJ Yamashita S Shai I Seidell J Magni P Waist Circumference as a vital sign in clinical practice: a consensus statement from the IAS and ICCR working group on visceral obesity. *Nat Rev Endocrinol.* (2020) 16:177–89. 10.1038/s41574-019-0310-7 32020062 PMC7027970

[31] Fang H Berg E Cheng X Shen W. How to best assess abdominal obesity. *Curr Opin Clin Nutr Metab Care.* (2018) 21:360–5. 10.1097/mco.0000000000000485 29916924 PMC6299450

[32] Jia WP Lu JX Xiang KS Bao YQ Lu HJ Chen L. Prediction of abdominal visceral obesity from body mass index, waist circumference and waist-hip ratio in Chinese adults: receiver operating characteristic curves analysis. *Biomed Environ Sci.* (2003) 16:206–11. 14631825

[33] Luo Y Ma X Shen Y Xu Y Xiong Q Zhang X Neck circumference as an effective measure for identifying cardio-metabolic syndrome: a comparison with waist circumference. *Endocrine.* (2017) 55:822–30. 10.1007/s12020-016-1151-y 27796813

[34] Arias Téllez MJ Martinez-Tellez B Soto J Sánchez-Delgado G. [Validity of neck circumference as a marker of adiposity in children and adolescents, and in adults: a systematic review]. *Nutr Hosp.* (2018) 35:707–21. 10.20960/nh.1582 29974783

[35] Atsawarungruangkit A Chenbhanich J Dickstein G. C-Peptide as a key risk factor for non-alcoholic fatty liver disease in the United States population. *World J Gastroenterol.* (2018) 24:3663–70. 10.3748/wjg.v24.i32.3663 30166861 PMC6113719

[36] Han X Xu P Zhou J Liu Y Xu H. Fasting C-Peptide is a significant indicator of nonalcoholic fatty liver disease in obese children. *Diabetes Res Clin Pract.* (2020) 160:108027. 10.1016/j.diabres.2020.108027 31958476

[37] Wang N Wang Y Zhang W Chen Y Chen X Wang C C-Peptide is associated with NAFLD inflammatory and fibrotic progression in Type 2 diabetes. *Diabetes Metab Res Rev.* (2020) 36:e3210. 10.1002/dmrr.3210 31351021

[38] Parlati L Régnier M Guillou H Postic C. New targets for NAFLD. *JHEP Rep.* (2021) 3:100346. 10.1016/j.jhepr.2021.100346 34667947 PMC8507191

[39] Negi CK Babica P Bajard L Bienertova-Vasku J Tarantino G. Insights into the molecular targets and emerging pharmacotherapeutic interventions for nonalcoholic fatty liver disease. *Metabolism.* (2022) 126:154925. 10.1016/j.metabol.2021.154925 34740573

